# Dendritic Cell-Based Immunotherapy in Hot and Cold Tumors

**DOI:** 10.3390/ijms23137325

**Published:** 2022-06-30

**Authors:** Byeong Hoon Kang, Heung Kyu Lee

**Affiliations:** Graduate School of Medical Science and Engineering, Korea Advanced Institute of Science and Technology (KAIST), Daejeon 34141, Korea; bhkang12@kaist.ac.kr

**Keywords:** dendritic cells, hot tumor, cold tumor, dendritic cell-based immunotherapy

## Abstract

Dendritic cells mediate innate and adaptive immune responses and are directly involved in the activation of cytotoxic T lymphocytes that kill tumor cells. Dendritic cell-based cancer immunotherapy has clinical benefits. Dendritic cell subsets are diverse, and tumors can be hot or cold, depending on their immunogenicity; this heterogeneity affects the success of dendritic cell-based immunotherapy. Here, we review the ontogeny of dendritic cells and dendritic cell subsets. We also review the characteristics of hot and cold tumors and briefly introduce therapeutic trials related to hot and cold tumors. Lastly, we discuss dendritic cell-based cancer immunotherapy in hot and cold tumors.

## 1. Introduction

Dendritic cells (DCs) were first discovered by Ralph M. Steinman in 1973 [1], and the importance of this discovery earned him the Nobel Prize in 2011 [2]. Much research has been performed to reveal the roles and mechanisms of DCs. When an abnormal reaction, such as infection, occurs in a host, DCs uptake antigens (Ags) from peripheral or central tissues via receptors and present these Ags using major histocompatibility complex (MHC) molecules. Then, DCs migrate to the lymphoid tissues and activate T cells. Thus, DCs mediate Ag sensing and T cell activation and are one of the most important cells in innate and adaptive immune responses [3].

Cancer is a disease that develops from normal cells by the accumulation of mutations of oncogenes and tumor-suppressor genes stimulated by stress or external stimuli [4]. Cancer rates have been increasing over time, and appropriate treatments are still being studied. Immunology has advanced considerably in recent years, and as the characteristics and roles of immune cells are revealed, the development of immunotherapies, which use immune cells, has been extensive [5].

Tumors can be hot or cold. The immunological characteristics of hot and cold tumors are different; they require different methods for targeting immunotherapies, and the immunotherapies have different efficacies [6]. DCs are mediators that directly stimulate T cell activation and present tumor Ags, so DCs have been studied for their use in cancer immunotherapy. However, there are various subsets of DCs, and tumors also have diverse immunological phenotypes. Thus, to treat tumors with DC-based immunotherapy, the characteristics of DCs and tumors need to be considered. In this review, we introduce the ontogeny and subsets of DCs. Then, we discuss tumor characteristics and review how DCs can be used as immunotherapy in hot and cold tumors.

## 2. Dendritic Cells

DCs present Ags. After DCs uptake Ags, they process and present them through MHC-I or MHC-II molecules. MHC-I molecules are expressed in most cells and present endogenous peptides from the cytosol; MHC-II molecules are only expressed on Ag-presenting cells (APCs) and present exogenous Ags. DCs have another mechanism of Ag presentation: cross-presentation that presents exogenous Ags on MHC-I molecules [7].

The functions of DCs are influenced by their maturation [3]. DCs express a range of pattern-recognition receptors (PRRs) internally and externally. These PRRs detect pathogen-associated molecular patterns (PAMPs); DCs can recognize Ags through PRRs and then mature in response [8,9]. Mature DCs need to physically contact T cells to present Ags to activate T cells. Soluble Ags can be easily delivered to T cells by lymphoid tissue-resident DCs. However, to deliver non-soluble Ags to T cells from peripheral tissues, DCs need to migrate to lymphoid tissues. The C-C chemokine receptor 7 (CCR7)-C-C motif chemokine ligand 19 (CCL19)/CCL21 axis is essential for migration from non-lymphoid tissue to lymphoid tissue via afferent lymphatic vessels [10]. After migration, only pathogen-exposed mature DCs can participate in T cell activation [3]. Mature DCs support T cell activation via co-stimulatory molecules in addition to stimulation of T cell receptor signaling. Without co-stimulatory molecules, DCs induce peripheral tolerance [11]. Activated T cells express CD40L, which promotes DC maturation. Thus, the relationship between T cells and DCs is bidirectional [3].

### 2.1. Ontogeny of Dendritic Cells

**Figure 1:**
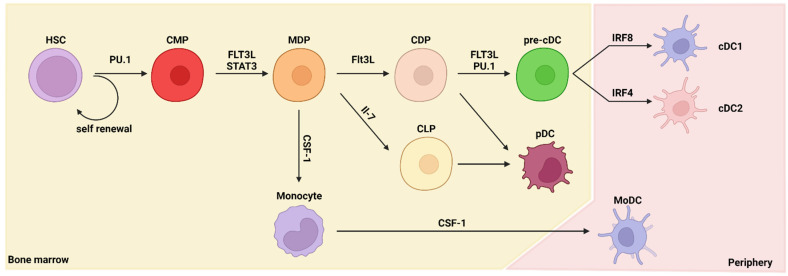
The ontogeny of dendritic cells. This figure summarizes the pathways leading to dendritic cell (DC) development in the bone marrow and peripheral tissue. Differentiation of DCs is dependent on multiple cytokines and transcription factors. HSC, hematopoietic stem cell; CMP, common myeloid progenitor; MDP, macrophage–DC progenitor; CDP, common DC progenitor; CLP, common lymphoid progenitor; pre-cDC, classical/conventional DC progenitor; cDC1, type 1 classical/conventional DC; cDC2, type 2 classical/conventional DC; pDC, plasmacytoid DC; MoDC, monocyte-derived DC; FLT3L, FMS-like tyrosine kinase 3 ligand; CSF-1, colony-stimulating factor 1; IRF, interferon-regulatory factor; STAT3, signal transducer and activator of transcription 3; IL, interleukin.

All immune cells are derived from hematopoietic stem cells (HSCs). HSCs do not proliferate frequently. Instead, their descendants, multipotent progenitors (MPPs), are highly proliferative [12]. HSCs are formed in the bone marrow and undergo hematopoiesis—differentiation into various progenitor cells. In particular, differentiation into cells of the myeloid lineage, such as DCs, is called myelopoiesis. The differentiation from HSCs to DCs requires several stages and several types of cytokines and transcription factors (Figure 1). The most important factor for DC differentiation is FMS-like tyrosine kinase 3 ligand (FLT3L). McKenna et al. reported that DC differentiation is FLT3L dependent using a *Flt3l*-knockout (KO) mouse model [13].

DC differentiation occurs in the bone marrow. First, HSCs differentiate into common myeloid progenitors (CMPs) and common lymphoid progenitors (CLPs). The PU.1 transcription factor has a major role in differentiation into CMPs [14]. Differentiation of DCs is inhibited in the absence of PU.1 expression, even with sufficient stimulation by FLT3L. Then, CMPs differentiate into macrophage–DC progenitors (MDPs) that are committed to the macrophage or DC lineages and not to the granulocyte lineage [15,16]. MDPs differentiate into common DC progenitors (CDPs), which further differentiate into pre-DCs. In the bone marrow, pre-DCs differentiate into plasmacytoid dendritic cells (pDCs) and classical/conventional dendritic cell progenitors (pre-cDCs), and pre-cDCs exit the bone marrow. Pre-cDCs differentiate into fully functioned DCs (cDC1s or cDC2s) in peripheral tissues [17,18,19,20]. Although pre-cDCs exit the bone marrow as progenitor cells, transcriptomics analysis has revealed that the lineage signatures of cDC1s and cDC2s are already primed in the bone marrow niche [21]. pDCs can be differentiated from both CMPs and CLPs [22,23,24]. DCs can be alternatively differentiated through a non-classical pathway; during inflammation or infection, DCs can also originate from monocytes in a CCR2-dependent manner and are known as monocyte-derived DCs (MoDCs) [25].

### 2.2. Dendritic Cell Subsets

**Figure 2:**
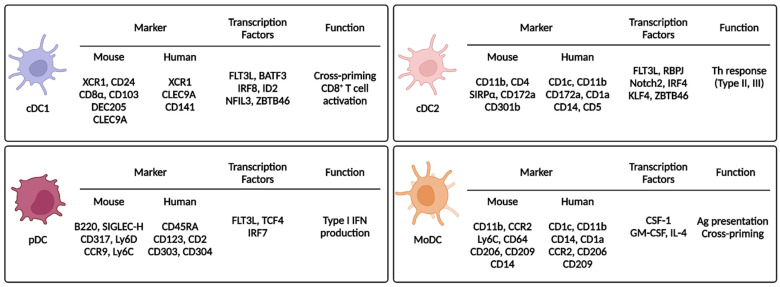
Dendritic cell subsets and their markers, transcription factors, and functions. This figure summarizes the characteristics of the DC subsets. Each DC subset expresses unique markers and transcription factors. XCR1, X-C motif chemokine receptor 1; CLEC9A, C-type lectin domain containing 9A; ID2, inhibitor of DNA binding 2; NFIL3, nuclear factor, interleukin 3 regulated; ZBTB46, zinc finger and BTB domain-containing 46; SIRPα, signal regulatory protein alpha; RBPJ, recombination signal binding protein for immunoglobulin kappa J region; KLF4, Krüppel-like factor 4; TCF4, transcription factor 4; GM-CSF, granulocyte-macrophage colony-stimulating factor; BATF3, basic leucine zipper ATF-like 3 transcription factor; SIGLEC, sialic acid-binding Ig-like lectin; Ly6, lymphocyte antigen 6; CCR, C-C chemokine receptor; IFN, interferon; Th, helper T; Ag, antigen.

DCs are classified as cDC1s, cDC2s, pDCs, or MoDCs. Differentiation of each subset requires various transcription factors and expression of unique markers (Figure 2).

#### 2.2.1. Type 1 Classical/Conventional Dendritic Cells

cDC1s are specialized in cross-presentation and require several key transcription factors. Hacker et al. used inhibitor DNA binding 2 (*Id2*) KO mice to show that ID2 affects the development of CD8α^+^ DCs and Langerhans cells and that transforming growth factor beta (TGF-β) contributes to ID2 activation [26]. Hildner et al. showed reduction of CD8α^+^ DCs in basic leucine zipper ATF-like 3 transcription factor (*Batf3*) KO mice, which resulted in impaired immunity against viral infections and cancer [27]. However, cDC1s can develop in *Batf3* KO mice mediated by interleukin (IL)-12 via a BATF- and BATF2-related compensatory mechanism [28]. In addition, interferon-regulatory factor (IRF) 8 (IRF8) and nuclear factor interleukin 3 regulated (NFIL3) are required for cDC1 development [29,30]. Recently, using a CRISPR-Cas9 genome editing system, cDC1 development was shown to be dependent on site-specificity of IRF8 [31]. Thus, cDC1 development involves many complicated transcription factor interactions, and more of these interactions remain to be discovered. The surface markers for mouse cDC1s are CD8α, CD103, XC chemokine receptor 1 (XCR1), C-type-lectin (CLEC) domain containing 9A (CLEC9A), and DEC205 [32,33,34]. The markers for human cDC1s are CD141, XCR1, CLEC9A, and DEC205 [35]. Both mouse and human cDC1s express XCR1, and XCR1 expression contributes to cDC1 chemotaxis via the XCR1/XC chemokine ligand 1 (XCL1) axis [3,36]. cDC1s in lymphoid tissue express CD8α, whereas cDC1s in non-lymphoid tissue and migratory cDC1s express CD103 [3]. cDC1s directly activate CD8^+^ T cells via cross-presentation of exogenous Ags using MHC-I [3,16]. Joke et al. injected irradiated *β2m*^−/−^ splenocytes loaded with ovalbumin. Although cell-associated ovalbumin was injected, the cytotoxic T lymphocyte (CTL) response occurred via the MHC-I receptors of CD8^+^ DCs [37], suggesting that exogenous Ags can be presented by MHC-I molecules through an unconventional pathway. cDC1s also activate CD8^+^ T cells indirectly via a type 1 T helper (Th1) response. Intracellular parasite infection induces Th1 immunity, and the main IL-12 producers during infection are BATF3-dependent CD103^+^ cDC1s or CD8^+^ cDC1s [38,39]. In addition to the contribution of cDC1s to adaptive immunity by T cell activation through Ag presentation, cDC1s express PRRs, including Toll-like receptor (TLR) 3 (TLR3) and TLR11 [39,40,41]. In cDC1s, TLR3 detects dsRNA of pathogens; then, TLR3 signaling upregulates the expression of cytokines and co-stimulatory molecules [40]. TLR11 in cDC1s detects intracellular parasites, and TLR11 signaling activates a Th1 response by stimulating secretion of IL-12 [39]. cDC1 cross-presentation and Th1 activation have been exploited in cancer immunotherapy by loading tumor-associated Ags (TAAs) into cDC1s [42]. In summary, cDC1s specialize in the activation of CTLs and a Th1 response.

#### 2.2.2. Type 2 Classical/Conventional Dendritic Cells

cDC2s are differentiated from pre-DCs through continuous stimulation of FLT3L [3,43], and this process requires several key transcription factors. Using genetically deficient mouse models, RelB [44], IRF2 [45], IRF4 [46,47], and recombination signal binding protein for immunoglobulin kappa J region (RBPJ) [48] have been identified as important factors for cDC2 development. RBPJ is a transcription factor involved in Notch signaling, so Notch signaling is important for cDC2 development. Notch2 and Krüppel-like factor 4 (KLF4) are also crucial for cDC2 development [49]. Although cDC1s express different markers depending on their location in lymphoid (CD8α^+^) or non-lymphoid (CD103^+^) tissue, cDC2s dominantly express CD11b regardless of location and also express CD4 [3,43,50]. Signal regulatory protein-α (SIRPα) and CD301b are also markers of cDC2s [49]. Endothelial cell-selective adhesion molecule (ESAM) is highly expressed in cDC2s in lymphoid tissues, and its expression is dependent on Notch2 signaling [51]. In humans, cDC2s express CD1a, CD1c, CD172a, and CLEC4C. Compared with human cDC1s, human cDC2s are more numerous and are therefore yet to be fully characterized [49]. cDC2s are involved in CD4^+^ T cell activation [3,43,50]. During a type 2 T helper (Th2) response, Tussiwand et al. showed that KLF4 is essential for cDC2 development, which is different from the essential Notch2 signaling pathway in cDC2s. The authors infected mice with pathogens to stimulate Th1, Th2, and Th17 responses and only the Th2 response was impaired. Thus, a house dust mite-induced asthma model was prevented in cDC2-specific KLF4-deleted mice [52]. In addition to a type II response, cDC2s also contribute to a type III response (Th17). Satpathy et al. used mice expressing zinc finger and BTB domain-containing 46 (ZBTB46) and green fluorescent protein (GFP) to show that intestinal cDC2s express CD103. They identified that CD103^+^CD11b^+^ cDC2s are dependent on Notch2 signaling, and DC-specific deletion of Notch2 decreases CD103^+^CD11b^+^ cDC2s. They demonstrated that CD103^+^CD11b^+^ cDC2s are essential for *Citrobacter rodentium* infection, which induces a Th17 response through IL-23 production [53]. cDC2s mainly activate CD4^+^ T cells, but some studies reported that cDC2s can also directly activate CD8^+^ T cells. Bosteels et al. reported that inflammatory cDC2s are induced under inflammatory conditions, and they share characteristics of both cDC1s and monocytes. Induction of inflammatory cDC2s activates CD8^+^ T cells more than cDC2s do [54]. Duong et al. also reported that cDC2s activate CD8^+^ T cells. They showed that type I interferon (IFN)-responsive cDC2s (IFN-stimulated genes [ISG^+^] DCs) externally express tumor-derived MHC class I molecules (“MHC-I dressing”), and MHC-I-dressed ISG^+^ DCs can activate CD8^+^ T cells [55].

#### 2.2.3. Plasmacytoid Dendritic Cells

pDCs were identified in 1999 by two research groups and were named for their plasmacytoid shape [56,57]. pDCs develop from pDC progenitors under continuous Flt3L stimulation, and transcription factor 4 (TCF4 [E2-2]) is the most important factor driving pDC differentiation. TCF4 also inhibits differentiation into the cDC lineage [58,59]. Then, transcription factor Spi-B restricts egress of non-dividing pDCs from the bone marrow until the pDCs are fully matured [60], when Runt-related transcription factor 2 (RUNX2) is activated. RUNX2 represses C-X-C chemokine receptor type 4 (CXCR4), which also inhibits egress from the bone marrow, and RUNX2 induces IRF7, which participates in terminal differentiation of pDCs [61]. pDCs express B220, sialic acid-binding Ig-like lectin H (SIGLEC-H), and CD317. In addition, CCR9 and lymphocyte antigen 6C (Ly6C) are also expressed in pDCs. Human pDCs also express CD45RA, CD123, and CD2 [50,62]. Although pDCs only make up 0.1–0.5% of the blood cells, pDCs are the main producer of type I IFN [62]. pDCs produce type I IFN by recognizing the viral genome during virus infection through endosomal TLRs. Compared with other immune cells, only pDCs have a high sensitivity for production of type I IFN, which is dependent on IRF7. When IFN-β is produced for the first time, IRF7 is induced by interferon alpha/beta receptor (IFNAR) signaling, and the IRF7/IFNAR axis forms a positive feedback loop. IFNAR signaling is not essential for pDC activity but is necessary to maintain their full functionality [62,63,64,65]. In relation to type I IFN production, TLRs expressed by pDCs primarily recognize pathogens, but pDCs also recognize infected cells. pDCs dock with infected cells using lymphocyte function-associated antigen-1 (LFA-1), and the TLR ligand is transferred to the pDCs from infected cells as an exosome or viral particle [62,66,67]. pDCs can also activate an adaptive immune response. Transcriptomics analysis of human peripheral blood mononuclear cells (PBMCs) enabled identification of Axl^+^ pDCs. Although Axl^+^ pDCs have a similar expression signature to pDCs, they produce less IFN-α in response to cytidine-phospho-guanosine and lipopolysaccharide stimulation than normal pDCs and activate CD4^+^ and CD8^+^ T cells more efficiently than normal pDCs [68,69,70]. Therefore, pDCs mainly protect the host by secreting type I IFN during viral infection; they also affect autoimmunity by acting on T cell and B cell responses. However, in a tumor environment, almost all pDC activity is suppressed by tumor-derived TGF-β [62].

#### 2.2.4. Monocyte-Derived Dendritic Cells

In general, conventional DCs are differentiated from pre-DCs. However, during injury or inflammation, DCs are differentiated from recruited monocytes rather than from DC progenitors, and the resulting DCs are known as inflammatory DCs or MoDCs. MoDCs migrate from the bone marrow to peripheral tissues in a CCR2-dependent manner. MoDCs express CD11b, Ly6C, and MHC-II, and human MoDCs express CD14 [3,42,43]. MoDCs are recruited to inflamed tissue during inflammation and are involved in Ag presentation and cytokine production [43,71]. Monocytes are rapidly recruited to a tumor site within 12 h after they recognize adenosine triphosphate (ATP) released from tumors. ATP promotes chemotaxis as well as differentiation of monocytes into MoDCs, and the MoDCs contribute to anti-tumor immunity by loading tumor Ags at the tumor site [72]. By exploiting this function, MoDCs have been used in cancer immunotherapy (i.e., DCVax^®^-L) [42,73].

## 3. Hot Tumors versus Cold Tumors

### 3.1. Characteristics

Solid tumors arise when normal cells become abnormally proliferative because of a mutation burden [4]. From the 1950s, Pierre Denoix began the development of the TNM system (T, the extent of primary tumor; N, the extent of regional lymph node metastasis; and M, the extent of distant metastasis) to classify tumors based on their anatomical extent and to give an indication of prognosis and treatment [74]. Tumors are composed of various cells, such as epithelial cells, fibroblasts, and immune cells, and the activation of immune cells, in particular, inhibits the growth of malignant cells [75]. In 2006, Galon et al. first used a tumor classification system for colorectal cancers by classifying type, density, and location of immune cells in colorectal cancers [76]. The authors conducted genomic analyses and in situ immunostaining and revealed that tumors from patients without recurrence and patients who survived had higher immune cell densities (especially of CD3^+^ and CD8^+^ immune cells). Thus, Galon et al. suggested that their first classification system based on immune cells in tumors was superior to and independent of the TNM system [76]. In the classification system of Galon et al., tumors are classified as two groups: hot (T cell-inflamed or T cell-infiltrated) and cold (not T cell-inflamed or T cell-infiltrated) [6,77]. In line with this immune-based classification, Camus et al. characterized colorectal cancers based on immune coordination. In addition to their previous study, the authors described four major profiles of colorectal cancers by their immune coordination (expression of CD3, CD8, granulysin, or IRF1) and angiogenesis (expression of vascular endothelial growth factor A [VEGF-A]) [78]. Galon et al. were the first to provide a measurement of the immune context by suggesting the use of a scoring system (immunoscore: I0–I4) [79], and they validated the immunoscore method in association with the TNM classification system [80]. Tumors can be classified based on this immune-context method [80], but some tumors have intermediate characteristics that are not accurately classified as hot or cold [78]. Furthermore, other characteristics, such as CD4^+^ T cells, B cells, cytokines, and tumor-intrinsic oncogenic pathways, affect cancer prognosis [6,81,82]. These intermediate tumors were classified as altered tumors, and altered tumors are divided into two subtypes: excluded and immunosuppressive [6].

In altered-excluded tumors, T cells are not absent but remain at the invasive margin. Tumors intrinsically hamper T cell infiltration to the central region. Oncogenic pathway of tumors correlate with T cells exclusion and β-catenin expression of tumors block the recruitment of DCs, which promotes T cells infiltration [83]. In addition, infiltration of T cells is blocked by a physical barrier. In the development of tumors, there are various factors to remodel tumor environment and promote metastasis. In particular, matrix metalloproteinases (MMPs) are highly expressed, and dysregulated matrix proteins act as a physical barrier. Furthermore, tumor-associated blood and lymphatics important to infiltration are also changed and induce a hypoxic environment, which promotes expression of the immunosuppressive hypoxia-inducible factor (HIF) family [84]. In altered-immunosuppressive tumors, on the other hand, infiltration to the center region is not absent but poor. Instead, infiltrated T cells are inactivated by various immunosuppressive factors and cells. First, immunosuppressive factors hamper immune cells activation. TGF-β, IL-10 and VEGF are representative factors of tumor environment. They directly or indirectly induce an immunosuppressive environment. Second, in the tumor environment, immunosuppressive cells inactivate immune cells. Myeloid-derived suppressor cells (MDSCs) express various factors to interfere with DCs differentiation and effector T cells. Another cell, regulatory T cells (Tregs), express immunosuppressive molecules and inhibitory receptors [85]. With these immunosuppressive environments, inhibitory receptors including programmed cell death protein 1 (PD-1), cytotoxic T lymphocyte-associated protein 4 (CTLA-4), and lymphocyte activation gene (LAG-3) are upregulated in tumor cells and APCs to further inhibit the T cell effector functions [6,85]. In the immunotherapy section of this review, we do not separate excluded and immunosuppressive tumor types but combine them into the cold tumor category.

In summary, there are various types of tumors (Table 1), and much research has been conducted to indicate prognosis and predict survival. Tumors can be classified and characterized based on immune context. Thus, we must consider the characteristics of tumors to treat and overcome them.

### 3.2. Overview of Immunotherapy for Hot and Cold Tumors

#### 3.2.1. Immunotherapy for Hot Tumors

The centers of hot tumors contain many infiltrated T cells, making T cells a main target for hot-tumor immunotherapy.

T cell co-inhibitory molecules, such as CTLA-4 and PD-1, are particularly important targets for immunotherapy. Immune checkpoint inhibitors (ICIs) can be used as immunotherapy to treat cancer by blocking these molecules. Such immunotherapies began with the development of the anti-CTLA-4 antibody (ipilimumab) by the U.S. Food and Drug Administration (FDA) in 2011; the anti-PD-1 antibody (nivolumab) was approved by the FDA in 2014 [86]. Combination therapy of anti-CTLA-4 and anti-PD-1 antibodies is effective in mouse models [87] and in patients with melanoma [88], renal cell carcinoma (RCC) [89], and lung cancer [90]. Other T cell co-inhibitory molecules include LAG-3, T cell immunoglobulin and mucin domain 3 (TIM-3), and T cell immunoglobulin and ITIM domain (TIGIT) [91]. Woo et al. showed that LAG-3 and PD-1 synergistically regulate T cell function and promote anti-tumor immunity in various mouse tumor models [92]. Sakuishi et al. demonstrated in 2010 that combination therapy of anti-TIM-3 antibody and anti-PD-1 ligand 1 (PD-L1) antibody in mice reduces CT26 colorectal carcinoma tumor size and increases IFN-γ^+^ tumor-infiltrating lymphocytes (TILs) [93]. In 2011, the therapeutic effect of anti-TIM-3 antibody and the importance of IFN-γ for T cells were validated [94]. Johnston et al. identified that TIGIT is highly expressed in human tumors and showed that tumors were reduced after treatment with anti-TIGIT antibody and anti-PD-L1 antibody in a mouse tumor model. The anti-tumor immunity was derived from CD8^+^ T cell activation [95]. Further studies reproduced the anti-tumor effect of TIGIT using human PBMCs [96,97]. These findings of efficacy of targeting other co-inhibitory molecules led to clinical trials of LAG-3 [98], TIM-3 [99], and TIGIT [100] in patients with cancer. In summary, many studies and trials have been conducted using ICIs to treat cancer.

T cells can also be activated using co-stimulatory molecules such as CD28, CD40L, and 4-1BB [91]. A clinical trial using co-stimulatory molecules to induce T cell activation has been conducted [101]. However, it is possible that T cell over-activation could result in excessive secretion of inflammatory cytokines. Indeed, risks of co-stimulatory molecule therapy were identified in several clinical trials [6]. Appropriate doses and administration time points have yet to be determined.

#### 3.2.2. Immunotherapy for Cold Tumors

Cold tumors have low or absent T cell infiltration and express high levels of immunosuppressive factors. To overcome these barriers to effective tumor eradication, cold tumors need to be converted to hot tumors. Here, we discuss several ways of achieving this goal.

First, cold tumors can be turned into hot tumors by recruitment of T cells into tumors by regulation of trafficking molecules. Nagarsheth et al. targeted C-X-C motif chemokine (CXCL) 9 (CXCL9) and CXCL10 and hypothesized that epigenetic regulation represses Th1 cytokines in cancer cells. They showed an increase in T cells, CXCL9 and CXCL10 when they inhibited PRC2, a methyltransferase of the Th1 cytokine H3K27 [102]. Another study targeted tumors more directly by injection of XCL1 into mouse tumors, which increased infiltration of cDC1s and Ag-specific CD8^+^ T cells [103]. Song et al. focused on a different route by injection of adeno-associated virus carrying VEGF-C into the cerebrospinal fluid (CSF) of a mouse model of glioblastoma, which increased lymphangiogenesis and induced efficient T cell priming. Injection of VEGF-C improved survival and had a synergistic effect when combined with immune checkpoint blockade therapy. Song et al. showed that glioblastoma, which originates in the brain, a tissue known to be resistant to immune checkpoint blockade (ICB) because of its immune privilege, can become a target of immune checkpoint blockade through regulation of trafficking [104].

Second, cold tumors can be converted to hot tumors and can increase therapeutic efficacy by blockade of pre-existing immunosuppressive and anti-inflammatory factors, such as signaling molecules, certain immune cells, and hypoxia. IL-10 and TGF-β are representative soluble factors that induce an immunosuppressive environment. Myeloid-derived suppressor cells and Tregs are also immunosuppressive. Sawant et al. demonstrated that IL-10 and IL-35 secreted from Tregs promoted expression of inhibitory receptors of TILs, and this mechanism was mediated by B lymphocyte-induced maturation protein 1 (BLIMP-1). Specific deletion of IL-10 in Tregs or reconstitution of IL-10 receptor deficient CD8^+^ T cells showed tumor reduction [105]. The colony-stimulating factor 1 (CSF-1)/CSF-1R axis is crucial for the mononuclear phagocyte system. In tumors, tumor-associated macrophages are also dependent on CSF-1R signaling for their survival. CSF-1R inhibitors inhibit immunosuppressive tumor-associated macrophages; ongoing clinical trials are testing CSF-1R inhibitors as a monotherapy or combination therapy [6,106]. Tumor microenvironment factors such as hypoxia can also be immune suppressive. We showed that glioblastoma is highly hypoxic compared with other tumors, and this hypoxic environment inhibits anti-tumor immunity. Thus, alleviating hypoxia enhanced survival in mice by increasing cytotoxicity of γδ T cells [107].

Lastly, cold tumors can be converted to hot tumors by priming the tumor microenvironment. Radiotherapy and chemotherapy that target tumor cells can cause inflammation around the tumor, which can induce tumor regression in tumors located in other parts of the body—the abscopal effect [6,108]. Zheng et al. showed the efficacy of radiotherapy in an ICI-resistant tumor model using local ionizing radiation coupled with vaccination and anti-PD-L1 antibody therapy. This approach increased CD8^+^ T cell infiltration by upregulation of CXCL10 and CCL5 and resulted in tumor regression [109]. Radiotherapy and chemotherapy induce immunogenic cell death of tumor cells, which can stimulate PRR via PAMP released from tumor cells. DNA released from dying tumor cells stimulates the cyclic GMP-AMP synthase (cGAS)-stimulator of interferon genes protein (STING) pathway. STING stimulation by tumor DNA is critical for IFN-β production in APCs in an IRF3-dependent manner, and IFN-β activates CD8^+^ T cells [110]. STING expression also correlates with favorable prognosis in human cancers, and intratumoral injection of a STING agonist normalizes vascular integrity, which promotes infiltration of effector CD8^+^ T cells [111]. Other PRRs (such as TLRs, retinoic acid-inducible gene 1-like receptors, and nucleotide-binding domain leucin-rich repeat-containing receptors) can be stimulated by damage-associated molecular patterns (DAMPs) released from tumors by activation of the immunogenic cell death pathway. PRR stimulation activates immune cells and contributes to anti-tumor immunity [6,108]. Thus, agonists of PRRs are currently being tested in clinical trials [108]. However, strategies to inflame tumor sites can damage normal cells as well as tumor cells. Therefore, oncolytic virus therapy, which specifically targets tumor cells, has been tested as an immunotherapy. Oncolytic viruses are genetically engineered for deletion of viral virulent factors. As oncogenic pathways of tumor cells often relate to viral replication, oncolytic viruses only replicate in tumor cells [112,113], which induces immunogenic cell death of tumor cells; DAMPs released from dying tumor cells activate PRRs. Oncolytic viruses can be further engineered to enhance immunity and promote their tropism. For example, cytokines such as granulocyte-macrophage colony-stimulating factor (GM-CSF), IL-12, and tumor necrosis factor (TNF) have been engineered to regulate immune cells, and co-stimulatory molecules are also expressed in engineered oncolytic viruses [112,113]. Furthermore, TAAs can be expressed by oncolytic viruses [112,113]. Other therapies have been developed that use engineered cells or peptides, such as chimeric antigen receptor (CAR) T cell and vaccine-based therapies [6,77,108].

In summary, there are numerous targets and therapies for hot and cold tumors. Although there are various methods of cancer immunotherapies, the principle to consider for an optimal therapeutic effect is simple: hot to hot and cold to hot.

## 4. Dendritic Cell-Based Cancer Immunotherapy

**Figure 3:**
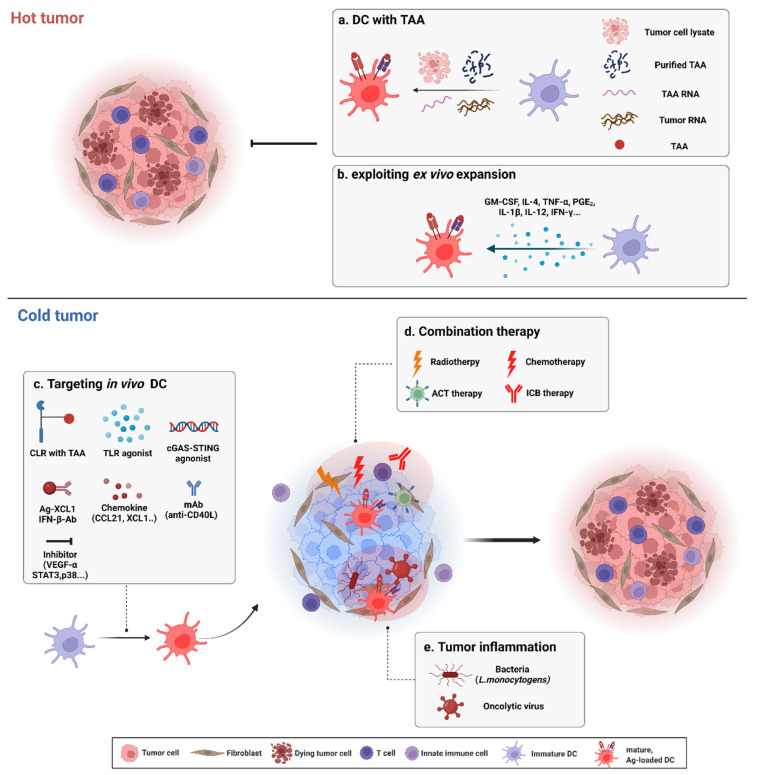
Dendritic cell (DC)-based immunotherapy for hot and cold tumors. Summary of targeting hot and cold tumors using DC-based immunotherapy is illustrated. (**a**) DC pulsed with various form of TAAs: irradiated tumor cell lysate, purified TAA peptide, purified TAA RNA and whole tumor RNA. (**b**) Various cytokines stimulate immature DC. For treatment of cold tumor, strategies to convert cold tumors to hot tumors are considered. (**c**) Direct binding of CLR-TAA, PRRs (TLR, STING...) agonist, chemokines, cytokines and monoclonal antibodies (mAbs) which bind co-stimulatory molecules can enhance migration and functionality of DC. Inhibition of tumor-derived immunosuppressive factors can reverse cold to hot. (**d**) DC-based immunotherapy combined with radio/chemotherapy, ACT therapy and ICB therapy increase the efficacy of DC therapy. (**e**) Bacteria or tumor-specific replicating virus induce inflammation in tumor which promotes immunologic hot phase. TAA, tumor-associated antigen; TNF-α, tumor necrosis factor-α; PGE_2_, prostaglandin E2; CLR, C-type lectin receptor; TLR, Toll-like receptor; mAb, monoclonal antibody; VEGF-α, vascular endothelial growth factor-α; ACT, adoptive cell transfer therapy; ICB, Immune checkpoint blockade.

DCs can activate CTLs via cross-presentation. CTLs act directly on tumor cells to achieve anti-tumor immunity. Thus, DCs can be used as a vaccine (DC vaccine) [2]. Traditionally, autologous DC-based cancer immunotherapy has been used. PBMCs from patients are extracted, differentiated, and expanded ex vivo to DCs, and immature or mature DCs are reinfused. Additionally, DCs can be pulsed with an irradiated tumor cell lysate and reinfused to induce a tumor-specific CTL response in patients [2]. TAAs specifically expressed in tumors can also be pulsed with DCs. DCs pulsed with TAAs or DCs that overexpress TAAs by mRNA transfection have activated CTL responses in patients. Ex vivo DC expansion increases the efficiency of DCs by influencing DC maturation; studies have been performed that added specific cytokines or other molecules to increase the efficiency of DC immunotherapy or that have considered the use of different DC subsets [2,114,115]. Cold tumors are more difficult to treat than hot tumors; thus, a diverse range of methods for DC-based immunotherapy for cold tumors has been developed [6,77,108] (Figure 3). For example, direct in vivo targeting of DCs has also been used, and methods for immunotherapy of cold tumors have been combined with DC therapy [115,116,117].

### 4.1. Classical Autologous Dendritic Cell-Based Cancer Immunotherapy

#### 4.1.1. Dendritic Cells with Tumor Cell Lysates

Albert et al. revealed the role of DCs in apoptosis. Only DCs can uptake apoptotic infected cells and cross-prime CD8^+^ T cells, which causes a CTL response [118]. Albert et al. also revealed the mechanism of DC uptake via α_v_β_5_ integrin and CD36 [119]. Similarly, apoptotic tumor cells, induced by irradiation, can be phagocytosed by and loaded by DCs using MHC molecules as a form of tumor-derived molecule or TAA. Irradiated tumor cell-derived molecules activate DC functions. In a clinical trial, DCs pulsed with tumor cell lysates from biopsies resulted in tumor regression in patients through CD8^+^ T cell activation [120]. In another clinical trial, patients with hepatocellular carcinoma received monthly boosters of a DC vaccine and gave clinical benefit [121]. A DC vaccine generated by exposure to tumor lysate also gave clinical benefit (improved survival) and increased tumor infiltration of CD8^+^ T cells in patients with glioma, a poorly immunogenic tumor type [122]. Initial results from patients with glioma that received DCVax^®^-L (an autologous DC vaccine generated from glioma lysate) in a large phase III clinical trial showed efficacy [73]; thus, an autologous DC vaccine generated from tumor lysate can be an efficient therapy for glioma. Another strategy that used an allogeneic tumor cell line instead of a biopsy from patients with melanoma also gave an increase in the CD8^+^ T cell response [123].

#### 4.1.2. Dendritic Cells with Tumor-Associated Antigens

CD8^+^ T cells recognize peptides presented by MHC-I molecules on APCs. Thus, TAAs are efficient targets to increase efficacy and specificity of a DC vaccine. In a mouse tumor model, bone marrow-derived dendritic cells or Langerhans cells pulsed with synthetic tumor peptides inhibited mouse tumor progression via a CTL response [124]. Several TAAs have been identified [42], and DC vaccines pulsed with TAAs are effective in several tumor types [2]. In a phase I study of advanced breast cancer, a p53-pulsed DC vaccine induced CTLs, and MRI scanning showed tumor regression [125]. Another research group targeted human telomerase reverse transcriptase (hTERT), which is highly expressed in human breast cancers, and demonstrated an increase in CD8^+^ T cell response [126]. Peptides loaded to DC vaccines are typically modified to increase their binding affinity to MHC molecules. Instead, Fong et al. focused on interactions with T cell receptors. The authors pulsed DCs with modified carcinoembryonic antigen (CEA), which alters T cell receptor interactions, and although CEA is poorly immunogenic, they were able to induce a durable CTL response and showed clinical benefit [127]. Another phase I trial studied the effect of pulsed DCs incubated with pan human leukocyte antigen (HLA) DR-binding protein to enhance CD4^+^ T cells activation, which induced immune responses in patients with metastatic RCC [128]. Instead of targeting TAAs, Kranz et al. directly targeted DCs in vivo. The authors used RNA-lipoplexes (RNA-LPXs) to protect RNA from extracellular nucleases. Intravenous administration of RNA-LPXs induced type I IFN production in DCs, and Ag-specific T cell responses were activated via type I IFN signaling. The authors were also the first to use systemic RNA-LPXs clinically, and they confirmed clinical efficacy in patients with cancer [129].

TAAs are a form of peptide, so it is possible that DCs transfected with TAA RNA can present TAAs extracellularly. DCs transfected with CEA RNA showed CEA-specific CTL response in vitro [130]. The same research group transfected DCs with TERT, and transfected DCs elicited a CTL response in vitro and in vivo [131]. Mice bearing mucin 1 (MUC1)-positive tumor cells were protected by MUC1-transfected DCs [132]. Although the MUC1-transgenic mice had immune tolerance to MUC1, IL-12 co-administration reversed this tolerance [132]. TAA-RNA transfected into DCs was also efficient in patients with prostate cancer [133]. Whole tumor RNA can also be a candidate for a transfected-DC vaccine. Heiser et al. isolated RNA from renal cancer tissue and transfected DCs with tumor RNA. The authors demonstrated that DCs transfected with tumor RNA also stimulated a tumor-specific CTL response in patients with RCC [134]. Indeed, DCs transfected with tumor RNA were more effective than DCs transfected with TAA-RNA [131].

The activity of DC vaccines pulsed with tumor cell lysates and exposed to TAAs (purified peptide or RNA transfection) is dependent on the DC vaccine itself. Several studies have shown that tumor cells are a continuous source of unaltered tumor Ags, and these Ags act like a tumor vaccine with DCs (i.e., causing DC–tumor cell fusion). For example, sorted CD11c^+^ DCs were electrofused with a melanoma cell line and injected into a mouse tumor model, and active immune responses were induced. In particular, immune responses induced by a DC–tumor hybrid were dependent on CD4^+^ and CD8^+^ T cells [135]. Zhou et al. mixed patients’ RCC cells with allogeneic DCs and resuspended the cells in polyethylene glycol for fusion. The authors confirmed that the DC–RCC hybrid stimulated CD4^+^ and CD8^+^ T cells to produce IFN-γ, and vaccination of patients with the DC–RCC hybrid resulted in decreased tumor size [136]. Akasaki et al. also fused DCs with glioma cells using polyethylene glycol; a DC–glioma hybrid inoculation of patients induced an Ag-specific CTL response [137].

#### 4.1.3. Exploiting Ex Vivo Expansion of Dendritic Cells

Ex vivo conditions, such as DC subsets, cytokines, and growth factors, should be taken into consideration to optimize autologous DC immunotherapy for tumors. Sallusot et al. reported that DCs can be differentiated ex vivo from PBMCs in media containing GM-CSF and IL-4 [138]. Other molecules are also used to supplement the medium to stimulate maturation and activation of DCs. TNF-α and prostaglandin E2 stimulate the maturation of DCs from immature DCs; in patients with melanoma, mature DCs pulsed with TAAs had superior induction of an immune response [139]. Immature DCs supplemented with IL-1β, TNF-α, IFN-γ, and polyinosinic:polycytidylic acid (poly I:C) induced the formation of mature cDC1-like DCs (αDC1s). αDC1s had increased IL-12 secretion, and vaccination of αDC1s pulsed with glioma Ags resulted in intensive infiltration of CD8^+^ T cells and CD68^+^ macrophages in the glioma region of patients [140]. In contrast, Wculek et al. sorted CD8^+^ DCs from FLT3L-stimulated mouse spleen. Then, they cultured CD8^+^ DCs in poly I:C and pulsed the cells with irradiated tumor cells. Injection of pulsed CD8^+^ DCs in tumor-bearing mice showed tumor regression via CD8^+^ T cell activation and had a synergistic effect with anti-PD-1 therapy [141]. Aside from conventional DC subsets, Tel et al. isolated pDCs from patients and pulsed pDCs with melanoma-associated Ags. Tel et al. were the first to identify that pulsed-pDC vaccination increased type I IFN, CD8^+^ T cells, and survival in patients with melanoma [142]. Most autologous DCs are generated by in vitro culture of monocytes. However, blood-circulating CD1c^+^ DCs can be isolated by cell sorting and used immediately. Vaccination with CD1c^+^ DCs briefly pulsed with tumor cells resulted in improved survival of prostate cancer patients [143] and metastatic melanoma patients [144].

### 4.2. Dendritic Cell-Based Cancer Immunotherapy in Cold Tumors

#### 4.2.1. Targeting the Pattern-Recognition Receptors of Dendritic Cells

DCs express a variety of PRRs, including C-type lectin receptors (CLRs) and TLRs. CLRs are unique markers of DCs. CLEC12A, a type of CLR broadly expressed in all human DC subsets, is efficiently internalized and endocytosed. Hutten et al. focused on this endocytic characteristic of CLEC12A. They conjugated a synthetic peptide to CLEC12A and showed that the conjugated peptide was efficiently cross-presented by DC subsets and resulted in CD8^+^ T cell activation [145]. Hahoud et al. also compared DEC205 and CLEC9A, other CLRs of DCs. Using a monoclonal antibody against ovalbumin, they demonstrated that targeting DEC205 and CLEC9A of CD8^+^ splenic DCs induced a response in OT-I T cells [146]. In humans, CDX1401 (human anti-DEC205 monoclonal antibody fused with NY-ESO1) treatment in patients with cancer generated immunity to NY-ESO1, and some patients showed clinical benefit [147]. CLRs can also be conjugated to other molecules to increase immunogenicity. To intensify immunogenicity only at the tumor site, CLEC9A conjugated to mutated IFN-α was used in several mouse tumor models, and strong immune responses occurred [148].

Cold tumors need to be converted to hot tumors for effective treatment. To boost or prime a cold tumor microenvironment, TLRs of DCs can be targeted for immunotherapy, which can be achieved by co-administration of a DC vaccine and a TLR agonist to stimulate T cell responses [149,150]. TLR agonists also increase intrinsic DC activity. Imiquimod, a TLR7 agonist, showed anti-tumor immunity in mice with skin tumors, even in *Rag2*^−/−^ mice that are deficient in mature T and B cells [151]. In particular, mast cells in the skin were responsive to imiquimod and upregulated CCL2. Increased CCL2 attracted pDCs to the skin tumor site, and recruited pDCs killed tumors directly by release or activation of cytotoxic effectors such as TNF-related apoptosis-inducing ligand (TRAIL), granzyme B (dependent on TLR7), and IFNAR signaling [151]. Furthermore, pDCs can affect adaptive immunity. In a mouse breast cancer model, tumor-associated pDCs were abundant but immunosuppressed in the presence of Tregs. Thus, depletion of pDCs inhibited tumor growth. However, intratumoral administration of a TLR7 agonist induced pDC activation and inhibited tumor growth through modulation of Tregs [152]. STING, another PRR, can prime a poorly immunogenic tumor microenvironment. Intratumoral administration of a STING agonist induced DC maturation and stimulated IFN-β release from APCs, which resulted in tumor regression [153]. Within T cell-inflamed but ICI-resistant cold tumors, local administration of a STING agonist stimulated APCs and reversed the resistant environment. Treatment with a STING agonist followed by anti-PD-1 therapy gave a synergistic effect [154]. The efficacy of TLR agonists can be otherwise maximized by modifying their form. For example, poly I:C encapsulated in biodegradable microparticles was able to be sustained in the lymph nodes, and intranodal injection of microparticles induced a slow spread of poly I:C. As a result, DCs were more activated [155]. Polymerization of a TLR agonist enhanced local retention and migration of APCs, which induced protective CD4^+^ and CD8^+^ T cell responses [156].

#### 4.2.2. Stimulation and Inhibition

Some patients with cold tumors and who have limited TILs or have TILs that localize at the edge of tumors fail to respond to ICI therapy. Salmon et al. analyzed Ag transport and investigated CD103^+^ DCs because they are uniquely able to transport Ags to tumor-associated lymph nodes. The authors injected FLT3L and identified accumulation and expansion of CD103^+^ DCs in the tumor mass. FLT3L injection followed by poly I:C promoted tumor regression in an ICI-resistant mouse tumor model and had a synergistic effect with anti-PD-L1 therapy [157]. Their study also showed that an immune response in an ICI-resistant tumor model was dependent on type I IFN [157]. The intrinsic tumor pathway also contributes to antibody resistance. However, specific delivery of IFN-β to the tumor site enhances antibody therapy, and linkage of antibody and IFN-β restores the function of DCs and causes dramatic regression of tumor growth [158]. Aside from cytokines, chemokine treatment also directly enhances the migration of DCs. Fusion of an Ag and XCL1 to create an XCL1-based vaccibody turned a poorly immunogenic tumor microenvironment into a highly immunogenic one. The XCL1-based vaccibody increased CD103^+^ DCs and the CD8^+^ T cell response [159]. In a clinical trial, DCs transduced with CCL21 using an adenoviral vector (Ad-CCL21-DC) were administrated to patients with lung cancer who had low infiltration of CD8^+^ T cells and were resistant to PD-1 therapy to attract T cells and DCs. Vaccination with Ad-CCL21-DC induced systemic Ag-specific immune responses and enhanced infiltration of CD8^+^ T cells into the tumor site [160]. As tumor PD-L1 expression was also upregulated, it is conceivable that Ad-CCL21-DC reverses ICI resistance and will be synergistic with ICI therapy.

DCs express other co-stimulatory molecules besides cytokines and chemokines, and stimulation of co-stimulatory molecules can be also an efficient method to reverse an immunosuppressive tumor microenvironment. CoAT (which includes an agonistic CD40 antibody, soluble Ag, and a TLR3 agonist) increased efficacy of DC immunization [161]. The CD40/CD40L axis is also important in an immunosuppressive tumor microenvironment. To overcome a cold tumor microenvironment, nitric oxide production by infiltrating myeloid cells (especially DCs that release TNF and nitric oxide) is essential. CD40/CD40L signaling strengthens Nitric oxide synthase 2 (NOS2) expression, and production of nitric oxide and TNF-α through CD40 contributes to tumor elimination by adoptive cell transfer [162]. Furthermore, NOS2, CD40, and TNF expression have a strong correlation with survival of patients with colorectal cancer [162]. In patients with RCC, treatment with MoDCs co-electroporated with tumor RNA and synthetic CD40L was well tolerated and resulted in immunologic responses coupled with extension of patient survival [163].

Inhibition of immunosuppressive factors also enhances the efficacy of DC-based therapy. In ovarian cancer, some patients were resistant to anti-PD-1 therapy because of tumor-soluble factors that block the migration of TILs to the tumor site. VEGF-A establishes a vascular endothelial cell barrier to prevent T cells homing to tumors [164]. Thus, bevacizumab, a VEGF-A-blocking antibody, had a synergistic effect with a DC-based vaccine in ovarian cancer patients [165]. Tumor-derived cytokines also directly inhibit the functionality of DCs. Haiyan et al. identified that tumor-associated cytokines induced signal transducer and activator of transcription 3 (STAT3) activity in DCs, and expression of STAT3 repressed *Id2* transcription required for DC-based anti-tumor immunity [166]. Using a conditional KO mouse model with ID2 overexpression in DCs, the authors corroborated that STAT3 deficiency and expression of ID2 in DCs enhanced the anti-tumor response. In particular, ID2 expression downregulated TNF-α, which induced proliferation of Tregs at the tumor site. As a result, there was a shift in the proportion of effector T cells to Tregs through ID2 expression in DCs, which reversed the poorly immunogenic tumor microenvironment [166]. Similarly, Yong et al. converted the proportion of effector T cells to Tregs by inhibition of p38 mitogen-activated protein kinase (MAPK) during DC differentiation [167]. The authors showed that inhibition of p38 in DCs upregulated OX40L and induced effector T cell proliferation in the presence of Tregs. They further showed that a p38 inhibitor repressed peroxisome proliferator-activated receptor gamma (PPARγ) and this downregulation induced expression of p50, the transcriptional activator of OX40L [167].

#### 4.2.3. Combination Therapy

Chemotherapy induces immunogenic cell death in tumor cells [6], and combination therapy together with immunogenic cell death increases DC functionality. Anthracycline-induced phosphorylation of eukaryotic translation initiation factor 2A (eIF2α) causes exposure of calreticulin in tumor cells, which acts as an “eat me” signal; thus, DCs phagocytose calreticulin-exposed tumor cells. Injection of recombinant calreticulin or blockade of eIF2α dephosphorylation increased immunogenicity of tumor cells and had a therapeutic benefit in tumor-bearing mice [168]. Vacchelli et al. identified single nucleotide polymorphisms that affected overall survival of chemotherapy-treated patients. They demonstrated that formyl peptide receptor 1, which recognizes annexin-1 in dying tumor cells, was affected only by single nucleotide polymorphisms in the formyl peptide receptor 1 (Fpr1) gene. Mutation of formyl peptide receptor 1 correlated with reduced survival, although the mutation of TLR3 and TLR4 were unaffected. During chemotherapy, activation of the FPR1/annexin-1 axis induced interactions with dying tumor cells and resulted in T cell activation, but migration of DCs to the tumor site was not affected by the activation of formyl peptide receptor 1 [169].

Chemotherapy also affects migration of DCs. Dying tumor cells treated with chemotherapy release ATP, which induces migration of CD11b^+^Ly6C^hi^ DCs to the tumor site [72]. Recruited CD11b^+^ DCs present TAAs to activate T cells. ATP also activates DCs via purinergic receptors. Using various KO mouse models, Ghiringhelli et al. demonstrated that the purinergic receptors in DCs activate the inflammasome of DCs and induce release of IL-1β, which further activates CD8^+^ T cells [170]. Chemotherapy also directly affects DCs. Platinum-based chemotherapy enhanced the T cell-stimulatory potential of DCs, but co-stimulatory molecules and cytokine production from DCs was not altered. Instead, co-inhibitory molecules, such as PD-L2, were significantly downregulated through STAT6 dephosphorylation [171].

Radiation therapy also induces immunogenic cell death in tumor cells. In particular, irradiated tumor cells are recognized by the cGAS-STING pathway in DCs. Activation of DCs via the STING pathway leads to secretion of IFN-β and activation of CD8^+^ T cells. Thus, treatment with exogenous IFN-β or a STING agonist promotes effective adaptive immune responses [172]. Photoradiation therapy-based DC vaccines induced tumor regression in mice with high-grade gliomas. Radiated high-grade gliomas released DAMP molecules and increased DC vaccine efficacy. Activation of DCs modified the ratio of T cells by increasing Th1 cells and decreasing Th17 cells, which resulted in an anti-tumor response [173].

Ineffective T cell responses can be reversed using monoclonal antibodies targeting PD-1 (anti-PD-1 antibody) and CD137 (anti-4-1BB antibody). However, Alfonso et al. showed that this therapy had a poor response in *Batf3*^−/−^ mice [174]. The authors suggested that the efficacy of ICI therapy is dependent on DCs, and ICI therapy in combination with a DC vaccine can induce a synergistic effect. In poorly immunogenic tumor models, the use of CTLA-4-blocking antibodies alone was not sufficient to improve patient survival/shrink tumor mass, but in combination with a DC vaccine, patients with melanoma had increased TILs, specific T cell responses, and clinical benefit in a pre-clinical trial [175]. An advanced form of a DC vaccine, GVAX (GM-CSF-transduced prostate cancer vaccine), gave clinical benefit and had a good safety profile when used in combination with ipilimumab (anti-CTLA-4 antibody) in a phase I dose-escalation trial [176]. Another form of DC vaccine, TriMix-DC (MoDCs electroporated with mRNA encoding CD70, CD40L, and TLR4), had a synergistic effect with ipilimumab in patients with advanced melanoma [177]. SD-101, a synthetic oligonucleotide with cytidine-phospho-guanosine motifs, stimulates pDCs through TLR9. Some patients with advanced melanoma who did not have pre-existing immune responses did not respond to anti-PD-1 monotherapy, but injection of SD-101 changed the tumor microenvironment to a hot tumor, which resulted in a CTL response [178]. In a mouse tumor model, CXCL9/10 recruited and activated CD8^+^ T cells. Anti-PD-1 therapy did not enhance the anti-tumor response in *Cxcr3*^−/−^ mice that were non-responsive to CXCL9/10. Epigenetic modulation to reverse the repression of CXCL9/10 in intratumoral CD103^+^ DCs reversed the efficacy of anti-PD-1 therapy [179]. The authors suggested that their pre-clinical data support activation of the CXCR3 system as a viable approach in anti-PD-1 therapy.

DC vaccines combined with adoptive cell transfer show clinical benefit. Patients with stage IV melanoma were treated with a DC vaccine followed by adoptive cell transfer, and this combination therapy was shown to be feasible with some patients experiencing tumor regression [180]. CCL22 is expressed in pancreatic cancer cells. Rapp et al. transduced T cells to overexpress CCR4 (the CCL22 receptor). Activation of the CCR4/CCL22 axis increased T cell recruitment and mediated interactions with intercellular adhesion molecule 1 in DCs. As a result, the CTL response was upregulated and mouse survival was enhanced [181]. Adoptive cell transfer increases the efficacy of DC-based immunotherapy. However, DCs also have an important role in adoptive cell transfer efficacy. Herranz et al. identified that vancomycin treatment with adoptive cell transfer inhibited mouse tumor growth. They revealed that the gut microbiota modulated migration of CD8^+^ DCs and affected IL-12 production in CD8^+^ DCs [182], which increased the efficacy of adoptive cell transfer.

The efficacy of DC vaccines can be enhanced when combined with modified bacteria or viruses. Killed but metabolically active *Listeria monocytogenes* were engineered to express tumor Ags. The modified *L. monocytogenes* activated MoDCs, and the Ags were successfully presented by MHC-I molecules on DCs. Targeting of in vivo DCs by modified *L. monocytogenes* induced the activation of CD4^+^ and CD8^+^ T cells and controlled mouse tumor growth [183]. Recently, Chen et al. incorporated cell membranes from *Escherichia. coli* and tumor-cell membranes in nanoparticles. They vaccinated tumor-bearing mice with the nanoparticles and showed improved activation of DCs and Ag-specific T cells. The nanoparticle vaccination enhanced mouse survival and provided a long-term anti-tumor effect [184]. Similar to stimulation by bacteria, tumor inflammation stimulated by a virus enhances the efficacy of DC vaccines. A hTERT-Ad was injected followed by a DC vaccine, and tumor-specific replicating virus infection induced oncolysis and tumor inflammation. Tumor inflammation induced by a virus provides all necessary signals to activate DCs and can induce greater anti-tumor responses compared with TLR activators [185]. It is suggested that combination therapy of a DC vaccine with an oncolytic virus is a good candidate for treatment of cold tumors.

#### 4.2.4. Targeting the Intrinsic Characteristics of Dendritic Cells

Although strategies to convert cold tumors to hot tumors and increase the Ags presentation ability of DC vaccines are successful, they are dispensable when migration of DCs is hampered. The intrinsic ability of DCs to migrate to a tumor site or a tumor-associated lymphoid site is essential to induce an anti-tumor immune response. Mitchell et al. identified that pre-conditioning with a tetanus/diphtheria toxoid increased migration of DCs to draining lymph nodes and promoted clinical benefit. Using a mouse model, the authors revealed that CD4^+^ T cells and CCL3 are essential for DC migration. They also demonstrated that CCL3 injection enhanced survival of tumor-bearing mice [186]. Intrinsic DC metabolism is influenced by metabolites in the tumor microenvironment, such as lipids and reactive oxygen species. In an ovarian cancer model, DCs migrated to the tumor site. However, recruited DCs were immunosuppressive, and tumor-bearing mice did not survive. In contrast to splenic DCs, intracellular peroxidized lipids formed in the presence of reactive oxygen species were abundant in tumor-associated DCs. Accumulation of lipids resulted in endoplasmic reticulum stress in tumor-associated DCs and led to expression of constitutive X-box binding protein 1 (XBP1), which disrupts lipid homeostasis in DCs. Thus, Cubillos-Ruiz et al. deleted *Xbp1* in DCs, which resulted in extended survival of ovarian cancer-bearing mice. Furthermore, they targeted *Xbp1* in tumor-associated DCs using polyethylenimine-based nanoparticles encapsulating siRNA and demonstrated therapeutic efficacy in an in vivo tumor model [187].

## 5. Conclusions

The development of DC-based anti-tumor immunotherapies is ongoing. Recently, single-cell RNA analysis has enabled the discovery of a novel DC subset called DC3 [70]. A variety of other factors and molecules related to DCs are also actively being studied. However, DC subsets are diverse, and tumors are heterogeneous and can be characterized as hot or cold by their immunogenicity, which makes their targeting a challenge. In highly immunogenic hot tumors, targeted T cell therapies and classical autologous DC-based therapies are efficient. In poorly immunogenic cold tumors, the conversion of cold tumors to hot tumors is a focus of much research. The priming method can convert a cold tumor microenvironment to a hot tumor microenvironment and increases the efficacy of DC vaccines. Ex vivo DCs, stimulatory agonists, combination therapy, and the targeting of in vivo DCs by specific receptors or TLRs to exploit intrinsic DC pathways have also been developed to convert a cold tumor microenvironment to a hot tumor microenvironment. In summary, DC-based cancer immunotherapy has been developed in many forms, and its efficacy has been enhanced. However, some considerations remain to be resolved, such as dosage, injection route, injection time points, and side effects, such as autoimmune reactions. Based on these considerations, thorough understanding of the characteristics of tumor and DC subsets will promote further development and increased efficacy of DC-based cancer immunotherapy.

## Figures and Tables

**Table 1:** Tumor classification based on immune context.

Classification	T Cell Infiltration	Immunoscore	Strategies for Immunotherapy
Hot tumor	High (center)	High	T cell targeting (targeting immune checkpoint)
Altered-excluded	Low (center) High (invasive margin)	Intermediate	T cell trafficking (chemokines, e.g., C-X-C motif chemokine 9/10/11) Inhibit physical barrier
Altered- immunosuppressive	Intermediate (center) Low (invasive margin)	Intermediate	Inhibitors of soluble immunosuppressive factors Immunosuppressive cells (e.g., myeloid-derived suppressor cells, regulatory T cells) Modulation of innate immune sensing
Cold	Absent	Low	Convert to hot (radiotherapy, chemotherapy) Adoptive cell transfer Oncolytic viruses Vaccine-based therapy

## Data Availability

Not applicable.
